# Comparative effectiveness of CDK4/6 inhibitor plus endocrine therapy combinations in HR-positive, HER2-negative metastatic breast cancer: the inspiration 01 study

**DOI:** 10.3389/fphar.2026.1757379

**Published:** 2026-04-13

**Authors:** Yan Jia, Jie Zhang, Xu Wang, Chunfang Hao, Chen Wang, Shufen Li, Ning Lu, Guolei Dong, Weipeng Zhao, Yongsheng Jia, Libin Huang, Jiangtao Gu, Yehui Shi, Zhongsheng Tong

**Affiliations:** 1 Department of Breast Oncology, Tianjin Medical University Cancer Institute & Hospital, Tianjin, China; 2 Tianjin’s Clinical Research Center for Cancer, Tianjin, China; 3 Key Laboratory of Breast Cancer Prevention and Therapy, National Clinical Research Center for Cancer, Tianjin Medical University, Ministry of Education, Tianjin, China; 4 Department of Medical Oncology, The First Hospital of Putian City, Putian, Fujian, China; 5 Department of Pharmacy, Tianjin Medical University Baodi Hospital, Tianjin, China

**Keywords:** cyclin-dependent kinase 4/6 inhibitor (CDK4/6i), endocrine therapy (ET), metastatic breast cancer (MBC), palbociclib, abemaciclib, dalpiciclib, HR-positive and HER2-negative (HR+/HER2-) breast cancer

## Abstract

**Background:**

The optimal combination of different cyclin-dependent kinase 4/6 inhibitors (CDK4/6i) with endocrine therapy (ET) for HR+/HER2− metastatic breast cancer (MBC) remains undefined due to a lack of head-to-head comparisons. This real-world study aimed to evaluate the effectiveness and safety of three CDK4/6 inhibitors combined with aromatase inhibitors (AI) or fulvestrant in the MBC setting.

**Methods:**

This study was a retrospective, observational, single-center analysis conducted in Tianjin Medical University Cancer Institute and Hospital, China between 1 January 2019 and 1 November 2023. The eligibility criteria were as follows: age ≥18 years; histologically confirmed hormone receptor-positive, HER2-negative breast cancer; recurrent or metastatic disease; at least one measurable lesion according to Response Evaluation Criteria in Solid Tumors (RECIST) version 1.1; and no prior systemic endocrine therapy for advanced disease. However, up to one line of prior chemotherapy in the metastatic setting was allowed. Statistical analyses were conducted using R software. Effective endpoints included progression-free survival (PFS), objective response rate (ORR), and clinical benefit rate (CBR).

**Results:**

This study enrolled 341 patients with HR+/HER2− MBC who received first-line CDK4/6i-based therapy consisting of palbociclib (n = 138), abemaciclib (n = 119), or dalpiciclib (n = 84) in combination with either an AI or fulvestrant. The median follow-up durations for PFS were 15.6 months, 10.9 months, and 18.2 months in the palbociclib, abemaciclib, and dalpiciclib groups, respectively. The maximum follow-up durations were 58.0 months for the palbociclib group, 53.7 months for the abemaciclib group, and 49.3 months for the dalpiciclib group. Regarding clinical benefit rate (CBR), the values for palbociclib, abemaciclib, and dalpiciclib combined with an AI versus fulvestrant were 93.8% (95% CI: 86.2%, 97.3%) versus 93.1% (83.6%, 97.3%), 97.7% (92.1%, 99.4%) versus 96.8% (83.8%, 99.4%), and 93.3% (84.1%, 97.4%) versus 87.5% (69.0%, 95.7%), respectively. For objective response rate (ORR), the corresponding rates were 37.5% (95% CI: 27.7%, 48.5%) versus 60.3% (47.5%, 71.9%), 48.9% (38.7%, 59.1%) versus 67.7% (50.1%, 81.4%), and 45.0% (33.1%, 57.5%) versus 54.2% (35.1%, 72.1%), respectively. Median PFS was 25.3 months for the palbociclib group, not reached (NR) for the abemaciclib group, and 36.0 months for the dalpiciclib group. Statistical analysis showed that both abemaciclib and dalpiciclib combinations were associated with longer PFS compared with palbociclib (both P < 0.05); however, due to the shorter median PFS follow-up duration and the lower number of PFS events in the abemaciclib group, the data for this group remain immature and warrant further follow-up. The PFS following CDK4/6i plus ET treatment was not significantly related to the status of key molecular biomarkers. The type of ET (AIs vs. fulvestrant) did not significantly affect PFS, although a consistent trend toward PFS benefit was observed in the fulvestrant-based combination group, without reaching statistical significance. Liver and bone metastases were associated with shorter PFS. Safety profiles were consistent with known spectra of each CDK4/6i, with no new signals identified.

**Conclusion:**

In this real-world analysis, dalpiciclib was associated with superior PFS compared to palbociclib as first-line CDK4/6i-based therapy for HR+/HER2- MBC. ET partner did not significantly impact effectiveness, supporting tailored CDK4/6i selection based on patient and disease characteristics.

## Introduction

Breast cancer represents the most commonly diagnosed malignancy and the leading cause of cancer-related mortality among women worldwide. In China, it has emerged as a major public health challenge, with incidence and mortality rates rising rapidly since the 1980s (Tao et al., 2023; Breast Cancer Expert Committee of National Cancer Quality Control Center, 2024). Breast cancer is highly heterogeneous, with hormone receptor-positive/human epidermal growth factor receptor 2-negative (HR+/HER2−) disease being the most prevalent subtype, accounting for approximately 60%–75% of all cases. Current therapeutic strategies for advanced or metastatic breast cancer aim to prolong survival and maintain or improve quality of life. However, overcoming therapy resistance and achieving a cure remain unmet clinical needs in this setting.

In recent years, cyclin-dependent kinase 4/6 (CDK4/6) inhibitors have revolutionized the management of advanced breast cancer. CDK4/6 play a critical role in regulating the G1 to S phase transition of the cell cycle. Accumulating clinical evidence has demonstrated that combining endocrine therapy (ET) with a CDK4/6 inhibitor (CDK4/6i) significantly improves progression-free survival (PFS) and overall survival (OS) in patients with HR+/HER2− metastatic breast cancer (MBC). In the PALOMA-2 trial, palbociclib—the first-in-class CDK4/6i—significantly extended median PFS compared to letrozole alone (27.6 months vs. 14.5 months) in postmenopausal women with previously untreated HR+/HER2− advanced breast cancer (Rugo et al., 2019). The PALOMA-3 study further showed that palbociclib plus fulvestrant improved median PFS compared to fulvestrant alone (9.5 months vs. 4.6 months) (Cristofanilli et al., 2016). Similarly, in the MONARCH-2 trial, abemaciclib combined with fulvestrant resulted in significantly longer PFS versus fulvestrant alone (median, 16.4 vs. 9.3 months) in patients whose breast cancer had progressed on prior ET (Sledge et al., 2017). Moreover, this combination provided a statistically significant and clinically meaningful OS benefit, with a median OS improvement of 9.4 months (46.7 vs. 37.3 months), irrespective of menopausal status (Sledge et al., 2020). The MONARCH-3 study also reported markedly prolonged median PFS with abemaciclib plus a nonsteroidal aromatase inhibitor (NSAI; anastrozole or letrozole) compared to placebo plus NSAI (28.18 months vs. 14.76 months) in postmenopausal women with treatment-naïve HR+/HER2− MBC (Johnston et al., 2019). In China, the phase 3 DAWNA-1 trial demonstrated that adding dalpiciclib to fulvestrant significantly improved PFS (15.7 months vs. 7.2 months) in patients with HR+/HER2− MBC who had progressed on prior ET (Xu et al., 2021). The subsequent DAWNA-2 trial showed a PFS benefit with dalpiciclib plus letrozole or anastrozole compared to ET alone (30.6 months vs. 18.2 months) in patients with untreated advanced HR+/HER2− breast cancer (Zhang et al., 2023). Based on this robust clinical evidence, CDK4/6 inhibitors combined with ET have been recommended as first-line therapy for HR+/HER2− MBC (Cancer Drug Clinical Research Committee of China Anti-Cancer Association, 2024). To date, four CDK4/6 inhibitors have been approved by the China National Medical Products Administration (NMPA): palbociclib (July 2018), abemaciclib (December 2020), dalpiciclib (December 2021), and ribociclib (January 2023). Although the MONALEESA trials demonstrated success with ribociclib, this agent was excluded from our analysis due to its recent approval in China.

The optimal combination of different CDK4/6 inhibitors with various endocrine agents—including aromatase inhibitors (AIs) and fulvestrant—as first-line endocrine treatment for HR+/HER2− MBC has not been clearly established. Given the absence of head-to-head comparisons between different CDK4/6i plus ET regimens, in this study, we analyzed real-world data to evaluate the effectiveness and safety of three CDK4/6 inhibitors (palbociclib, abemaciclib, and dalpiciclib) combined with three aromatase inhibitors (letrozole, anastrozole, and exemestane) or fulvestrant as initial endocrine therapy in patients with HR+/HER2− MBC. The primary objective was to identify the most effective partners to optimize the treatment strategies for this patient population. Although the combination of CDK4/6i with ET achieved significant clinical success in advanced HR+/HER2− breast cancer, however, several challenges remained including identifying patients most likely to benefit prior to treatment initiation and determining the optimal timing and sequencing of therapy (Morrison et al., 2024). Thus, we also tested the biomarkers such as ER, PR, HER2, Ki67, TILs and et al. to evaluate the association between the status of predictive biomarker and the efficacy of CDK4/6i-based combination therapy.

## Materials & methods

### Design and treatment

This single-center retrospective study was designed to evaluate the antitumor activity, safety, and tolerability of three CDK4/6 inhibitors—palbociclib, abemaciclib, and dalpiciclib—in combination with endocrine therapy, which included either aromatase inhibitors (letrozole, anastrozole, or exemestane) or fulvestrant, in patients with HR+/HER2− MBC. Potential biomarkers were further evaluated to determine their predictive value for the effectiveness of combining three CDK4/6 inhibitors with endocrine therapy. From 1 January 2019, to 1 November 2023, eligible patients from Tianjin Medical University Cancer Institute & Hospital were required to meet the following criteria: age 18 years or older; histologically confirmed hormone receptor-positive, HER2-negative breast cancer; recurrent or metastatic disease; at least one measurable lesion as defined by Response Evaluation Criteria in Solid Tumors (RECIST) version 1.1 (Eisenhauer et al., 2009); and no prior systemic endocrine therapy for advanced disease, although up to one line of chemotherapy in the metastatic setting was permitted.

Patients received standard treatment with a CDK4/6 inhibitor (palbociclib, abemaciclib, or dalpiciclib) combined with an aromatase inhibitor or fulvestrant until disease progression or unacceptable toxicity. There were no restrictions regarding prior neoadjuvant or adjuvant therapies administered in the early breast cancer setting. Since up to one line of chemotherapy was allowed in the metastatic setting, enrolled patients included those who had not received any systemic therapy for advanced disease, as well as those who experienced disease progression or achieved disease control following first-line chemotherapy and were subsequently switched to a CDK4/6 inhibitor combined with ET. In cases of clinical partial response or stable disease, patients with initial radiologic evidence of progression were allowed to continue treatment pending a confirmatory scan performed approximately 2 months later to verify disease progression. The real-world data was derived from the Electronic Medical Record (EMR) System of Tianjin Medical University Cancer Institute and Hospital between 1 January 2019 and 1 November 2023. The EMR system captured comprehensive clinical information, including patient demographics, diagnoses, laboratory test results, medication prescriptions, and procedure records. All personal identifiers were removed and replaced with unique study IDs before analysis. A series of quality control procedures were applied to ensure reliability. The study was approved by the institutional review boards and ethics committees at Tianjin Medical University Cancer Institute and Hospital. Data acquisition, validation and quality control were performed at Tianjin Medical University Cancer Institute and Hospital with appropriate informed consent procedures.

### Effectiveness and safety

Effective and survival data, including ORR, CBR, PFS and OS were analyzed in this study. Patients evaluable for treatment response were those with measurable disease at baseline who received at least one dose of a CDK4/6 inhibitor plus endocrine therapy and had at least one post-baseline tumor assessment, or those who discontinued therapy before the first scheduled scan due to progressive disease (PD) or a treatment-related adverse event (AE).

Safety was assessed based on AEs graded according to the Common Terminology Criteria for Adverse Events (CTCAE), version 5.0, during treatment and for up to 30 days after treatment discontinuation. Serious AEs were recorded for up to 90 days following the last dose of combination therapy. To ensure patient safety, laboratory tests, vital signs, and physical examinations were routinely monitored. Tumor imaging was performed every 3 months, and treatment response was evaluated per RECIST v1.1 by experienced radiologists.

### Statistical analysis

Demographics and disease characteristics were summarized by treatment arm. Descriptive statistics (median, IQR and range) were presented for continuous variables, and counts and percentages were presented for categorical variables. Progression-free survival (PFS) and overall survival (OS) were estimated using the Kaplan-Meier method. PFS was defined as the time from the beginning of treatment to the first occurrence of disease progression or death from any cause (whichever occurs first). Participants without disease progression or death at the time of the data cutoff were censored at the date of the last progression-free disease assessment. OS was defined as the time from the beginning of treatment to death from any cause. Participants who had not died at the time of the data cutoff were censored at the last date they were known to be alive. DFS was defined as the time from radical surgery to any recurrence or metastasis. The univariate cox-proportional hazards model with the Efron method of handling ties was used to estimate the HR (hazard ratio) and its 95% confidence interval (CI) between treatment groups; p-values were derived from the log-rank test. Additionally, to account for potential imbalances between treatment groups and adjust for key demographic and disease characteristics, multivariable Cox regression model was employed for key comparisons. Covariates included age and menopausal status at the start of CDK4/6i plus ET, *de novo* stage IV status, prior chemotherapy (neoadjuvant, adjuvant, or first-line), prior adjuvant endocrine therapy, the presence of visceral metastases, and the type of endocrine therapy (AI vs. fulvestrant). Adjusted hazard ratios, 95% confidence intervals, and corresponding p-values were presented. Given the retrospective design, limited sample size, and exploratory nature of this study, no formal hypothesis testing was prespecified. All analyses were hypothesis-generating; therefore, nominal p-values were reported without adjustment for multiplicity.

Objective response rate (ORR) was defined as the proportion of participants with a best overall response of complete response (CR) or partial response (PR). Clinical benefit rate (CBR) was defined as the proportion of participants with a best overall response of CR or PR or stable disease (SD) lasted for a minimum of 6 months. Participants without post-baseline objective response assessments were counted as non-responders. Confidence intervals for ORR and CBR were calculated using the Wilson score method. Odds ratios (ORRs) were calculated using unconditional maximum likelihood estimation, with 95% confidence intervals derived via Wald normal approximation. To explore the association between ORR and treatment, nominal p-values were calculated using the Chi-square tests. The CBR was compared between groups using Fisher’s exact test to account for small expected cell frequencies.

All statistical analyses described above were conducted using R software, version 4.3.3 (R Foundation for Statistical Computing, Vienna, Austria).

## Results

### The characteristics of patients

**FIGURE 1:**
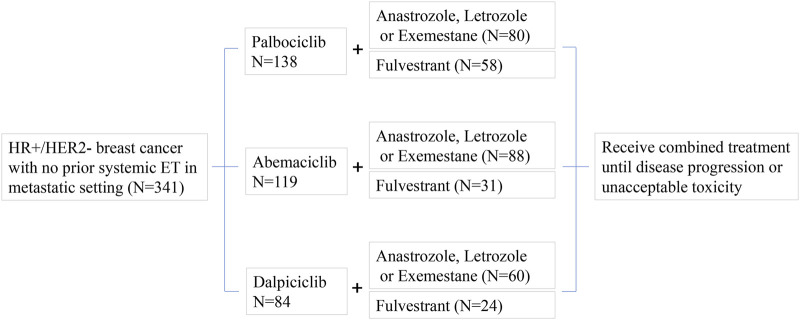
Study design and patient allocation for the treatment of HR+/HER2-metastatic breast cancer in patients with no prior systemic endocrine therapy (N = 341). Patients were administered one of three CDK4/6 inhibitors—palbociclib (N = 138), abemaciclib (N = 119), or dalpiciclib (N = 84)—in combination with either an aromatase inhibitor (anastrozole, letrozole, or exemestane) or fulvestrant. Treatment was continued until disease progression or the occurrence of unacceptable toxicity.

As illustrated in Figure 1, patients was enrolled to evaluate the antitumor activity, safety, and tolerability of three CDK4/6 inhibitors—palbociclib, abemaciclib, and dalpiciclib—in combination with endocrine therapy, which included either aromatase inhibitors (letrozole, anastrozole, or exemestane) or fulvestrant, in patients with HR+/HER2− MBC. From 1 January 2019, to 1 November 2023, a total of 341 female patients were enrolled in this study. The follow-up duration for patients receiving CDK4/6 inhibitors combined with endocrine therapy was as follows: for palbociclib, it ranged from 3.0 to 58.0 months; for abemaciclib, from 3.0 to 53.7 months; and for dalpiciclib, from 3.0 to 49.3 months. The median follow-up durations for the palbociclib, abemaciclib, and dalpiciclib groups were 26.2 months, 11.3 months, and 24.3 months, respectively. All tumors were confirmed as HR+/HER2-breast cancer, with no prior systemic endocrine therapy for advanced disease. Baseline patient characteristics were summarized in Table 1. All patients had an Eastern Cooperative Oncology Group (ECOG) performance status of 0 or 1. The median age was 56 years (range: 32–81), 55 years (range: 29–86), and 59 years (range: 34–87) in the palbociclib, abemaciclib, and dalpiciclib plus endocrine therapy (ET) groups, respectively. All participants were female. Menopausal status was comparable across the three CDK4/6 inhibitor (CDK4/6i) treatment groups, with the majority being postmenopausal (69.6%, 64.7%, and 76.2%, respectively). Invasive ductal carcinoma was the predominant pathological type, accounting for 91.3%, 93.3%, and 97.6% of patients in the palbociclib, abemaciclib, and dalpiciclib plus ET groups, respectively. Other pathological types included invasive lobular carcinoma and invasive micropapillary carcinoma. Most tumors were histological grade 2, comprising 86.2%, 89.1%, and 83.4% across the three groups.

**TABLE 1:** Baseline of patient information and clinical characteristics.

Characteristics	Palbociclib + ET (N = 138)	Abemaciclib + ET (N = 119)	Dalpiciclib + ET (N = 84)
Age at initial diagnosis of BC^a^, years			
Mean (SD)	51.4 (12.17)	50.3 (11.87)	53.3 (12.00)
Median	51	48	54
Min, max	27, 76	23, 86	27, 87
Age categories at initial diagnosis, no. (%)			
≤35	13 (9.4)	9 (7.6)	6 (7.1)
35–65	102 (73.9)	95 (79.8)	62 (73.8)
≥65	23 (16.7)	15 (12.6)	16 (19.1)
Age at treatment of CDK4/6i plus ET, years			
Mean (SD)	55.9 (12.25)	55.3 (11.76)	57.8 (11.72)
Median	56	55	59
Min, max	32, 81	29, 86	34, 87
Age categories at treatment of CDK4/6i plus ET, no. (%)			
≤35	5 (3.6)	4 (3.4)	3 (3.6)
35–65	95 (68.9)	91 (76.4)	55 (65.4)
≥65	38 (27.5)	24 (20.2)	26 (31.0)
Gender/Female			
No. (%)	138 (100.0)	119 (100.0)	84 (100.0)
Menstrual status when treated with CDK4/6i plus ET, no. (%)			
Premenopausal	42 (30.4)	42 (35.3)	20 (23.8)
Postmenopausal	96 (69.6)	77 (64.7)	64 (76.2)
Left or right breast cancer at initial diagnosis, no. (%)			
Left	72 (52.2)	62 (52.1)	47 (56.0)
Right	66 (47.8)	57 (47.9)	37 (44.0)
Pathological type, no. (%)			
Invasive ductal carcinoma	126 (91.3)	111 (93.3)	82 (97.6)
Invasive lobular carcinoma	9 (6.5)	3 (2.5)	2 (2.4)
Invasive micropapillary carcinoma	11 (8.0)	17 (14.3)	2 (2.4)
Others	9 (6.5)	10 (8.4)	0 (0.0)
Histological grade, no. (%)			
1	4 (2.9)	0 (0.0)	7 (8.3)
2	119 (86.2)	106 (89.1)	70 (83.4)
3	15 (10.9)	13 (10.9)	7 (8.3)
The expression of ER ^b^ , no. (%)			
1%–10%	1 (0.7)	0 (0.0)	0 (0.0)
≥10%	137 (99.3)	119 (100.0)	84 (100.0)
The expression of PR ^c^ , no. (%)			
<1%	31 (22.5)	26 (21.8)	8 (9.5)
1%–10%	16 (11.6)	16 (13.5)	15 (17.9)
≥10%	91 (65.9)	77 (64.7)	61 (72.6)
HER2 status, no. (%)			
0	44 (31.9)	34 (28.5)	33 (39.3)
1+	56 (40.6)	51 (42.9)	29 (34.5)
2+, FISH-	38 (27.5)	34 (28.6)	22 (26.2)
The expression of Ki67, no. (%)			
≤20%	57 (41.3)	51 (42.9)	40 (47.6)
>20%	81 (58.7)	68 (57.1)	44 (52.4)
The expression of P53, no. (%)			
<10%	82 (59.4)	54 (45.4)	37 (44.1)
≥10%	32 (23.2)	37 (31.1)	19 (22.6)
NA^d^	24 (17.4)	28 (23.5)	28 (33.3)
The expression of EGFR, no. (%)			
<2%	82 (59.4)	62 (52.1)	36 (42.9)
≥2%	12 (8.7)	3 (2.5)	2 (2.3)
NA^d^	44 (31.9)	54 (45.4)	46 (54.8)
The expression of AR ^e^ , no. (%)			
<10%	5 (3.6)	6 (5.0)	8 (9.5)
≥10%	71 (51.4)	55 (46.2)	29 (34.5)
NA^d^	62 (45.0)	58 (48.8)	47 (56.0)
The status of TILs ^f^ , no. (%)			
<5%	29 (21.0)	27 (22.7)	18 (21.5)
≥5%	27 (19.6)	24 (20.2)	17 (20.2)
NA^d^	82 (59.4)	68 (57.1)	49 (58.3)
Family history, no. (%)			
Yes	31 (22.5)	16 (13.4)	14 (16.7)
Preliminary TNM stage, no. (%)			
I	24 (17.4)	15 (12.6)	10 (11.9)
II	50 (36.2)	48 (40.3)	23 (27.4)
III	40 (29.0)	32 (26.9)	25 (29.8)
IV	24 (17.4)	24 (20.2)	26 (30.9)
T stage, no. (%)			
1	43 (31.2)	30 (25.2)	27 (32.1)
2	85 (61.6)	71 (59.7)	47 (56.0)
3	6 (4.3)	11 (9.2)	8 (9.5)
4	4 (2.9)	7 (5.9)	2 (2.4)
N stage, no. (%)			
0	50 (36.2)	36 (30.3)	26 (31.0)
1	35 (25.4)	38 (31.9)	27 (32.1)
2	26 (18.8)	17 (14.3)	10 (11.9)
3	27 (19.6)	28 (23.5)	21 (25.0)
M stage^g^, no. (%)			
0	114 (82.6)	95 (79.8)	58 (69.1)
1	24 (17.4)	24 (20.2)	26 (30.9)
Treatment on early stage of BC, no. (%)			
Neoadjuvant chemotherapy	31 (22.5)	21 (17.6)	12 (14.3)
Surgery	112 (81.2)	95 (79.8)	57 (67.9)
Adjuvant chemotherapy	99 (71.7)	81 (68.1)	49 (58.3)
Adjuvant radiotherapy	52 (37.7)	51 (42.9)	33 (39.3)
Adjuvant endocrine therapy	100 (72.5)	92 (77.3)	52 (61.9)
Metastasis, no. (%)			
Liver	27 (19.6)	14 (11.8)	15 (17.9)
Lung	41 (29.7)	27 (22.7)	24 (28.6)
Bone	68 (49.3)	53 (44.5)	41 (48.8)
Brain	3 (2.2)	4 (3.4)	1 (1.2)
Lymph node	62 (44.9)	54 (45.4)	35 (41.7)
Thoracic wall	17 (12.3)	18 (15.1)	9 (10.7)
Pleura and pleural effusion	14 (10.1)	23 (19.4)	15 (17.8)
Skin and soft tissue	2 (1.4)	3 (2.5)	4 (4.8)
Others	6 (4.3)	3 (2.5)	5 (6.0)
First-line chemotherapy, no. (%)			
Yes	30 (21.7)	60 (50.4)	16 (19.0)
No	108 (78.3)	59 (49.6)	68 (81.0)
Partners of CDK4/6 inhibitors, no. (%)			
Aromatase inhibitors	80 (58.0)	88 (73.9)	60 (71.4)
Anastrozole	19 (13.8)	20 (16.8)	20 (23.8)
Letrozole	33 (23.9)	48 (40.3)	33 (39.3)
Exemestane	28 (20.3)	20 (16.8)	7 (8.3)
Fulvestrant	58 (42.0)	31 (26.1)	24 (28.6)
Follow-up time, months			
Median	26.2	11.3	24.3
Min, max	3.0, 58.0	3.0, 53.7	3.0, 49.3

a. BC, breast cancer; b. ER, estrogen receptor; c. PR, progesterone receptor; d. NA, no available data; e. AR, androgen receptor; f. TILs, tumor-infiltrating lymphocytes; g. M stage, metastatic status at initial diagnosis.

Regarding molecular markers, only one patient in the palbociclib plus ET group had low estrogen receptor (ER) expression (defined as ER 1%–10%). Progesterone receptor (PR) expression varied, with low PR expression (PR 1%–10%) observed in 11.6%, 13.5%, and 17.9% of patients, and negative PR expression in 22.5%, 21.8%, and 9.5% of patients in the palbociclib, abemaciclib, and dalpiciclib groups, respectively. HER2-low status (defined as immunohistochemistry [IHC] score of +1 or +2 with negative fluorescence *in situ* hybridization [FISH]) was observed in 68.1%, 71.5%, and 60.7% of patients across the three treatment groups; the remaining patients had HER2-negative breast cancer. The distribution of Ki67 expression was similar across the three CDK4/6 inhibitor plus endocrine therapy (CDK4/6i + ET) groups, with Ki67 levels exceeding 20% in 58.7%, 57.1%, and 52.4% of cases, respectively. Expressions of P53, epidermal growth factor receptor (EGFR), androgen receptor (AR), and tumor-infiltrating lymphocytes (TILs) status are detailed in Table 1.

With respect to family history and prior treatments, 22.5%, 13.4%, and 16.7% of patients reported a family history of malignancy (at least one relative with one type of cancer). According to the tumor-node-metastasis (TNM) staging system, *de novo* stage IV breast cancer was present in 17.4%, 20.2%, and 30.9% of patients in the three CDK4/6i (palbociclib, abemaciclib, or dalpiciclib) plus ET groups, respectively. Detailed T, N, and M stage information was provided in Table 1. In this study, 82.6%, 79.8%, and 69.1% of patients were initially diagnosed at an early stage and had opportunity to receive neoadjuvant or adjuvant therapy. Among these, 81.2%, 79.8%, and 67.9% underwent surgery, while 72.5%, 77.3%, and 61.9% received adjuvant endocrine therapy (tamoxifen or aromatase inhibitors). Neoadjuvant chemotherapy was administered to 22.5%, 17.6%, and 14.3% of patients, respectively. No patient received neoadjuvant endocrine therapy. The comparable DFS across all three groups indicated a well-balanced distribution of the major prognostic factor at baseline (showed in Supplementary Figure S1).

In terms of metastatic sites, bone and lymph nodes were the most common locations. Bone metastasis was present in 49.3%, 44.5%, and 48.8% of patients, and lymph node metastasis in 44.9%, 45.4%, and 41.7% across the three groups (palbociclib, abemaciclib, or dalpiciclib plus ET groups). Visceral metastases included lung (29.7%, 22.7%, and 28.6%) and liver (19.6%, 11.8%, and 17.9%). Other metastatic sites involved the thoracic wall, pleura and pleural effusion, brain, skin, and soft tissue.

In the metastatic setting, up to one line of prior chemotherapy was permitted before initiating CDK4/6i combined with ET. Prior first-line chemotherapy for advanced disease was reported in 21.7%, 50.4%, and 19.0% of patients in the three groups (palbociclib, abemaciclib, or dalpiciclib plus ET groups), respectively. Taxanes and anthracyclines were the most commonly used prior chemotherapeutic agents. As shown in Figure 1, aromatase inhibitors (anastrozole, letrozole, or exemestane) were administered in combination with CDK4/6i in 58.0%, 73.9%, and 71.4% of patients; the remaining patients received CDK4/6i plus fulvestrant in the three groups. All patients continued combination therapy until disease progression or unacceptable toxicity.

### Antitumor activity

Effectiveness was evaluated in all patients who received at least one dose of CDK4/6 inhibitor (CDK4/6i) in combination with endocrine therapy (ET), had measurable disease at baseline according to RECIST v1.1, and either underwent at least one post-baseline imaging assessment or discontinued treatment due to progressive disease or a treatment-related adverse event prior to the first scan. Effective endpoints, including objective response rate (ORR) and clinical benefit rate (CBR) were summarized for this study.

**FIGURE 2:**
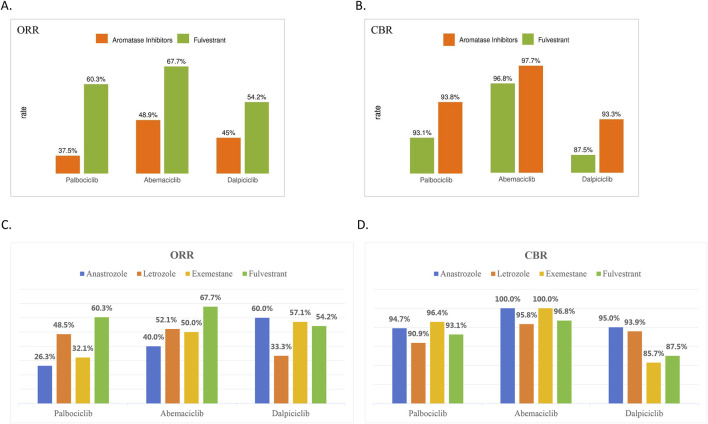
Effective outcomes of CDK4/6 inhibitors combined with endocrine therapy in HR+/HER2-metastatic breast cancer. **(A)** Objective response rate (ORR) and **(B)** clinical benefit rate (CBR) for palbociclib, abemaciclib, and dalpiciclib combined with aromatase inhibitor or fulvestrant. **(C)** ORR and **(D)** CBR for palbociclib, abemaciclib, and dalpiciclib when administered with specific endocrine agents (anastrozole, letrozole, exemestane, or fulvestrant). Data are presented as percentages.

In the metastatic setting, patients were treated with various combination regimens. In the study, endocrine agents included aromatase inhibitors (letrozole, anastrozole, exemestane) and fulvestrant, while CDK4/6 inhibitors consisted of palbociclib, abemaciclib, and dalpiciclib. Both the ORR and CBR at the data cut-off date were shown in Figure 2.

As shown in Figure 2A, the objective response rates (ORR) for the three CDK4/6 inhibitors—palbociclib, abemaciclib, and dalpiciclib—when combined with endocrine therapy (aromatase inhibitors versus fulvestrant) were 37.5% (95% CI: 27.7%, 48.5%) vs. 60.3% (47.5%, 71.9%), 48.9% (38.7%, 59.1%) vs. 67.7% (50.1%, 81.4%), and 45% (33.1%, 57.5%) vs. 54.2% (35.1%, 72.1%), respectively. Regarding the ORR, the odds ratio (OR) for palbociclib plus endocrine therapy (ET) versus abemaciclib plus ET was 0.77 (95% CI: 0.47, 1.25); for palbociclib plus ET versus dalpiciclib plus ET, the OR was 0.98 (95% CI: 0.57, 1.69); and for abemaciclib plus ET versus dalpiciclib plus ET, the OR was 1.28 (95% CI: 0.73, 2.24).

Additionally, as shown in Figure 2B, the clinical benefit rate (CBR) for the same regimens were 93.8% (86.2%, 97.3%) vs. 93.1% (83.6%, 97.3%), 97.7% (92.1%, 99.4%) vs. 96.8% (83.8%, 99.4%), and 93.3% (84.1%, 97.4%) vs. 87.5% (69.0%, 95.7%), respectively. Regarding the CBR, the odds ratio for palbociclib plus endocrine therapy (ET) versus abemaciclib plus ET was 0.37 (95% CI: 0.10, 1.40); for palbociclib plus ET versus dalpiciclib plus ET, it was 1.30 (95% CI: 0.47, 3.64); and for abemaciclib plus ET versus dalpiciclib plus ET, it was 3.52 (95% CI: 0.88, 14.01).

As illustrated in Figure 2C, the ORR for palbociclib combined with anastrozole, letrozole, exemestane, or fulvestrant were 26.3% (95% CI: 11.8%, 48.8%), 48.5% (32.5%, 64.8%), 32.1% (17.9%, 50.7%), and 60.3% (47.5%, 71.9%), respectively. The corresponding ORR values for abemaciclib-based combinations were 40.0% (21.9%, 61.3%), 52.1% (38.3%, 65.5%), 50.0% (29.9%, 70.1%), and 67.7% (50.1%, 81.4%), while those for dalpiciclib combinations were 60.0% (38.7%, 78.1%), 33.3% (19.8%, 50.4%), 57.1% (25.0%, 84.2%), and 54.2% (35.1%, 72.1%), respectively. Thus, when combined with either palbociclib or abemaciclib, fulvestrant was associated with a higher ORR compared to aromatase inhibitors. In contrast, among dalpiciclib-based regimens, the combination with letrozole yielded a relatively lower ORR than the other three endocrine agents. No significant differences were observed among the groups (P > 0.05).

As shown in Figure 2D, the CBR for palbociclib in combination with anastrozole, letrozole, exemestane, or fulvestrant were 94.7% (75.4%, 99.1%), 90.9% (76.4%, 96.9%), 96.4% (82.3%, 99.4%), and 93.1% (83.6%, 97.3%), respectively. The corresponding CBR values for abemaciclib combinations were 100.0% (88.1%, 100%), 95.8% (86.0%, 98.8%), 100.0% (88.1%, 100%), and 96.8% (83.8%, 99.4%), and for dalpiciclib combinations were 95.0% (76.4%, 99.1%), 93.9% (80.4%, 98.3%), 85.7% (48.7%, 97.4%), and 87.5% (69.0%, 95.7%), respectively. The palbociclib and abemaciclib groups exhibited similar trends in CBR; however, abemaciclib-based combinations overall achieved more favorable CBR outcomes. Among the dalpiciclib combination groups, therapy with exemestane or fulvestrant resulted in lower CBR. No significant differences were observed among the groups (P > 0.05).

### Survival

**FIGURE 3:**
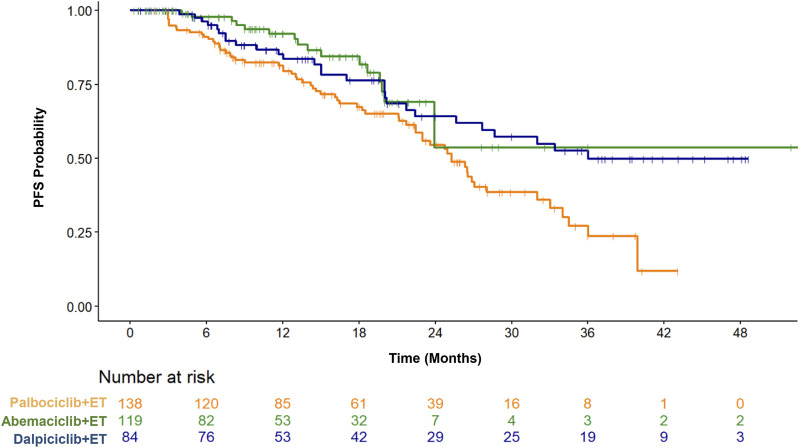
Kaplan-Meier curves for progression-free survival (PFS) in patients with HR+/HER2-metastatic breast cancer treated with palbociclib, abemaciclib, or dalpiciclib in combination with endocrine therapy (ET). The number of patients at risk at each time point is provided below the graph.

1. Progression-free survival (PFS) was analyzed in this study, as of the data cut-off date (1 November 2023), 65, 17, and 28 PFS events were recorded in the palbociclib, abemaciclib, and dalpiciclib combination groups, respectively. Responders remained on study and continued combined therapy beyond the data cut-off. All patient subgroups derived benefit from CDK4/6i plus ET, which delayed the initiation of second-line chemotherapy. As of the data cut-off date, the median PFS for the palbociclib, abemaciclib, and dalpiciclib groups was 25.3 months, not reached (NR), and 36.0 months, respectively. The median PFS follow-up durations for the three CDK4/6 inhibitor groups are 15.6 months, 10.9 months, and 18.2 months, with a pooled median follow-up of 14 months across all groups (Figure 3).

In this study, multivariable Cox proportional hazards analyses were conducted, adjusting for key clinical variables including age, menopausal status, *de novo* stage IV disease, prior chemotherapy, prior adjuvant endocrine therapy, visceral metastases, and type of endocrine therapy (aromatase inhibitor versus fulvestrant). Pairwise comparisons for PFS were performed using multivariable Cox regression. After adjustment for these clinical variables, pairwise comparisons among the three CDK4/6 inhibitor (CDK4/6i) plus endocrine therapy (ET) regimens indicated that PFS was comparable between the abemaciclib and dalpiciclib groups (dalpiciclib + ET vs. abemaciclib + ET, p = 0.454; HR [95% CI]: 1.36 [0.61–3.02]). Furthermore, both the abemaciclib and dalpiciclib groups demonstrated longer PFS than the palbociclib group (abemaciclib + ET vs. palbociclib + ET, p = 0.0426, HR [95% CI]: 0.54 [0.30–0.98]; dalpiciclib + ET vs. palbociclib + ET, p = 0.0396, HR [95% CI]: 0.61 [0.38–0.98]). Given the relatively small number of PFS events (17 events out of 119 cases) in the abemaciclib combination group, even after statistical adjustments, further follow-up data are still required to draw a definitive conclusion regarding its superiority over the palbociclib combination regimen.

**FIGURE 4:**
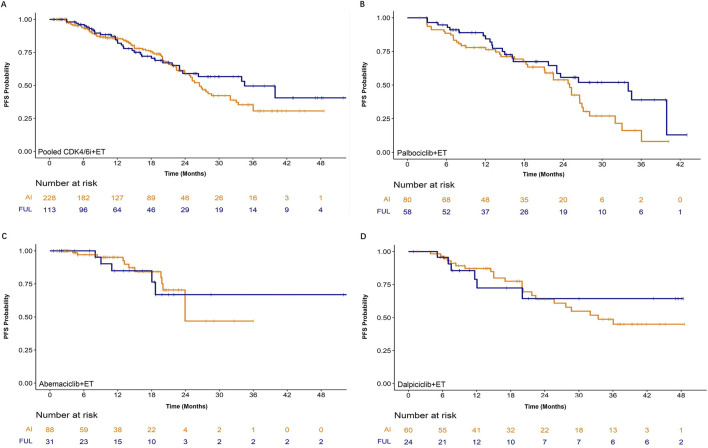
Kaplan-Meier curves for progression-free survival (PFS) comparing the effectiveness of different endocrine therapy (ET) backbones (aromatase inhibitor, AI; fulvestrant, FUL) in combination with **(A)** pooled CDK4/6 inhibitors, **(B)** abemaciclib, **(C)** palbociclib, and **(D)** dalpiciclib in patients with HR+/HER2-metastatic breast cancer. The number of patients at risk at each time point is shown below the corresponding panel.

As shown in Figure 1 and Table 1, 42.0%, 26.1%, and 28.6% of metastatic breast cancer (MBC) patients in the palbociclib, abemaciclib, and dalpiciclib groups, respectively, received first-line fulvestrant combined with CDK4/6i due to prior aromatase inhibitor (AI) treatment in the early breast cancer setting. Clinically, fulvestrant was administered to patients who had previously received adjuvant aromatase inhibitors (AIs) and were therefore deemed unsuitable for AI re-challenge in the metastatic setting. This population also included individuals with prior adjuvant treatment failure—often considered a “harder-to-treat” group—who similarly received fulvestrant. Thus, in this study, the impact of ET type (AI vs. fulvestrant) on PFS was further evaluated (Figure 4). Results indicated that CDK4/6i plus ET prolonged PFS regardless of the specific endocrine agent (aromatase inhibitor or fulvestrant) used. No significant difference in PFS was observed between patients receiving CDK4/6i plus AI or fulvestrant (all P > 0.05). In the palbociclib group, fulvestrant demonstrated a tendency towards a positive response of PFS benefit relative to AI, though this was not found to be statistically significant (HR [95% CI]:0.63 [0.37, 1.04], P = 0.0659).

**FIGURE 5:**
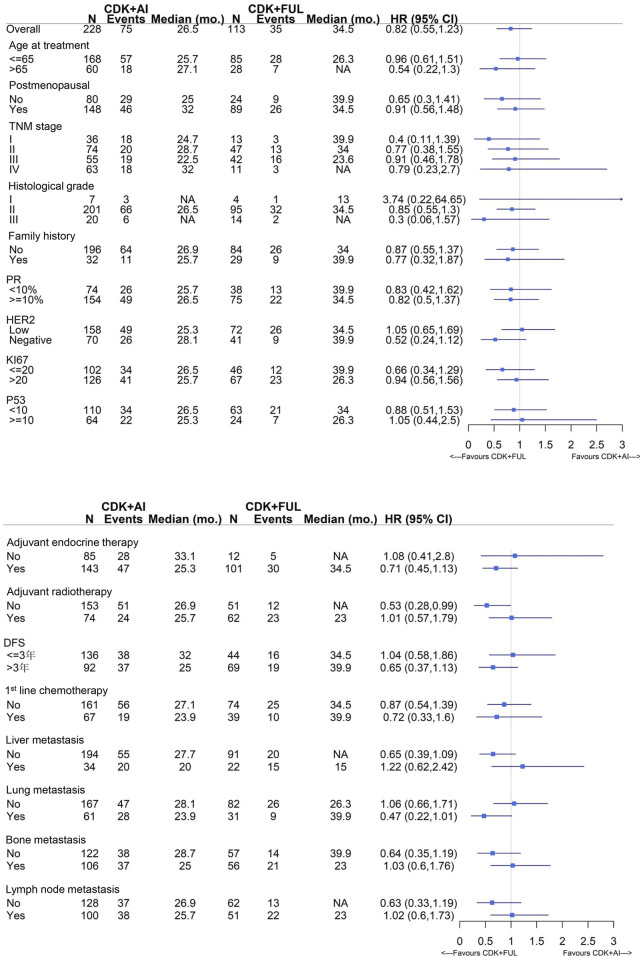
Forest plot of the subgroup analysis for patients treated with CDK4/6 inhibitors plus endocrine therapy (AIs versus fulvestrant), illustrating PFS benefit across different subgroups. CDK, CDK4/6 inhibitor, AI, aromatase inhibitor; FUL, fulvestrant.

In clinical practice, when combining CDK4/6 inhibitor therapy, the choice between an AI and fulvestrant was often based on the patient’s prior treatment history and efficacy assessments during early-stage therapy. For patients assessed as having AI resistance, fulvestrant-based combination therapy was generally preferred. In this study, we therefore evaluated the benefit from CDK4/6i combined ET (AI versus fulvestrant) based on various factors. Within the current sample size, a consistent trend toward progression-free survival (PFS) benefit was observed in the fulvestrant-based combination therapy group, although this difference did not reach statistical significance (Figure 5). Pooled analysis of fulvestrant versus aromatase inhibitors, after adjusting for key demographic and baseline disease characteristics, yielded a hazard ratio (HR) of 0.66 (95% CI: 0.42, 1.03) for fulvestrant compared with aromatase inhibitors, with a corresponding p-value of 0.0675. Given the limited sample size and number of events, multivariable adjustment was not applied in subgroup analyses to avoid model overfitting and instability of coefficient estimates.

**FIGURE 6:**
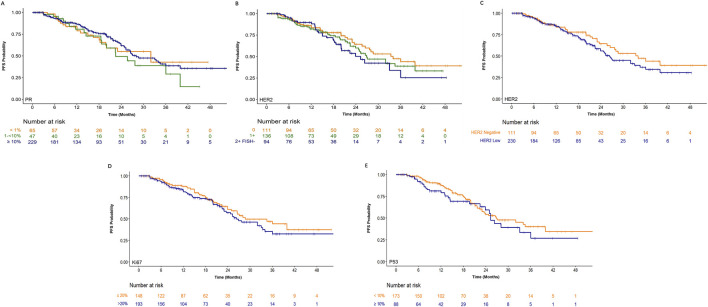
Kaplan-Meier curves for progression-free survival (PFS) in patients with HR+/HER2- metastatic breast cancer (all P > 0.05), stratified by specific molecular signature (PR, HER2, Ki67 and P53). The number of patients at risk in each subgroup over time is listed below each panel. **(A)** Stratified by high (>10%), low (1–10%), or negative (<1%) progesterone receptor (PR) expression; **(B)** Stratified by HER2 expression level (IHC 0, IHC 1+, or IHC 2+ with FISH negative); **(C)** Stratified by HER2 status (HER2-negative vs. HER2-low expression); **(D)** Stratified by Ki67 (cut-off 20%); **(E)** Stratified by p53 (cut-off 10%).

All patients had pathologically confirmed HR+/HER2– MBC. The association between treatment effectiveness and molecular features, including HER2 expression, hormone receptor (HR) status, Ki67 level, and P53 status, was evaluated in the study. As illustrated in Figure 6 and Table 2, PFS did not significantly differ among patients with high (>10%), weak (1%– 10%), or negative (<1%) progesterone receptor (PR) expression. Similarly, HER2 status (negative vs. low expression) showed no significant correlation with PFS. Although a trend toward improved PFS was observed in HER2-negative breast cancer compared to HER2-low cases, this did not reach statistical significance. Neither Ki67 (cut-off 20%) nor P53 (cut-off 10%) expression related to PFS. In summary, no significant association was found between PFS following CDK4/6i plus ET treatment and HER2 expression, Ki67 level, PR status, or P53 status (all P > 0.05). Due to limited data, only one patient had weak ER expression (1%–10%), and few had data on EGFR, AR, or tumor-infiltrating lymphocytes (TILs), the comparisons based on these markers were not feasible. Thus, within this small cohort, no significant associations were observed between treatment response (PFS) to CDK4/6i plus ET and the specific molecular biomarkers examined.

**TABLE 2:** The association between factors and PFS.

Factors	Comparison	HR (95% CI)	P-value (log-rank)
Age when treated	35–65 vs. ≤35	0.66 (0.27, 1.64)	0.3692
	>65 vs. ≤35	0.55 (0.21, 1.44)	0.2282
	>65 vs. 35–65	1.26 (0.8, 1.98)	0.3192
Postmenopausal when treated	Yes vs. no	0.77 (0.52, 1.14)	0.1973
*De novo* stage IV	1 vs. 0	0.65 (0.4, 1.05)	0.0756
Tumor grade	III vs. I	1.09 (0.32, 3.7)	0.8866
	II vs. I	1.44 (0.53, 3.94)	0.4742
	III vs. II	0.93 (0.45, 1.92)	0.8529
1L chemotherapy (palbociclib)	Yes vs. no	0.62 (0.32, 1.19)	0.1421
1L chemotherapy (abemaciclib)	Yes vs. no	3.22 (1.05, 9.88)	0.0315
1L chemotherapy (dalpiciclib)	Yes vs. no	1.93 (0.72, 5.19)	0.1816
Adjuvant radiotherapy	Yes vs. no	1.58 (1.08, 2.31)	0.0173
Adjuvant endocrine therapy	Yes vs. no	1.19 (0.79, 1.79)	0.4144
Liver metastasis	Yes vs. no	3.21 (2.14, 4.81)	0.0001
Bone metastasis	Yes vs. no	1.5 (1.03, 2.19)	0.0342
Lung metastasis	Yes vs. no	1.1 (0.74, 1.63)	0.6490
Lymph node metastasis	Yes vs. no	1.42 (0.97, 2.07)	0.0669
PR	≥10% vs. 1%–10%	0.75 (0.45, 1.26)	0.2735
	≥10% vs. <1%	0.91 (0.56, 1.49)	0.7201
	1%–10% vs. <1%	1.23 (0.65, 2.31)	0.5110
HER2	2+, FISH - vs. 1+	1.16 (0.73, 1.83)	0.5368
	2+, FISH - vs. 0	1.44 (0.88, 2.35)	0.1442
	1+ vs. 0	1.21 (0.78, 1.9)	0.3918
HER2	Low vs. negative	1.3 (0.87, 1.94)	0.2043
Ki67	>20% vs. ≤20%	1.23 (0.84, 1.8)	0.2833
P53	≥10% vs. <10%	1.29 (0.82, 2.02)	0.2718
Family history of cancer	Yes vs. no	0.81 (0.5, 1.31)	0.3854

In the TNM staging system, primary tumor size, regional lymph node involvement, and distant metastasis are established risk and prognostic factors in breast cancer. In the palbociclib, abemaciclib, and dalpiciclib groups, 17.4%, 20.2%, and 32.1% of patients, respectively, had *de novo* stage IV disease; the remainder underwent surgical resection of primary tumors and axillary or sentinel lymph nodes during early-stage disease. As shown in Supplementary Figures S2A–D; Table 2, TNM stage, primary tumor status, lymph node metastasis, and distant organ metastasis were not significantly associated with PFS (all P > 0.05). Additional factors, including tumor grade, age or menopausal status at treatment, and family history, were also analyzed, but none showed a statistically significant relationship with PFS (all P > 0.05, Supplementary Figures S2E–H; Table 2).

**FIGURE 7:**
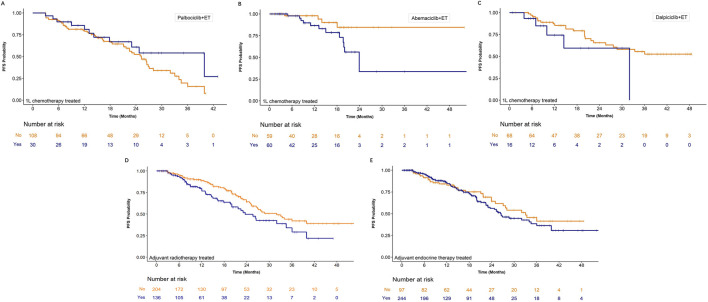
Subgroup analyses of progression-free survival (PFS) based on prior treatment history. Kaplan-Meier curves for patients treated with palbociclib plus endocrine therapy (ET), abemaciclib plus ET, and dalpiciclib plus ET are stratified by prior **(A–C)** chemotherapy, **(D)** adjuvant radiotherapy, and **(E)** adjuvant endocrine therapy. The corresponding number of patients at risk at each time point is provided below each panel.

Prior to CDK4/6i plus ET, 21.7%, 50.4%, and 19.0% of patients in the palbociclib, abemaciclib, and dalpiciclib groups, respectively, had received first-line chemotherapy. As shown in Figures 7A,C, prior chemotherapy did not affect PFS in the palbociclib or dalpiciclib groups (P > 0.05, Table 2). However, in the abemaciclib group, patients without prior first-line chemotherapy exhibited significantly longer PFS (P = 0.0315, Figure 7B; Table 2). Adjuvant radiotherapy during early-stage breast cancer was associated with poorer PFS following CDK4/6i plus ET (P = 0.0173, Figure 7D; Table 2). In contrast, prior adjuvant endocrine therapy did not influence PFS (P > 0.05, Figure 7E).

**FIGURE 8:**
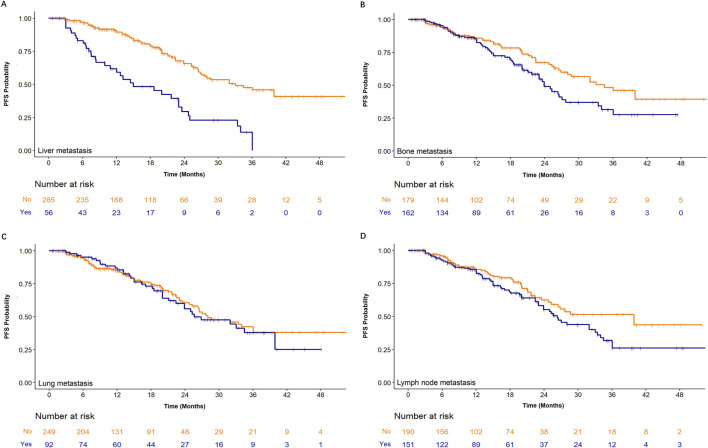
Subgroup analyses of progression-free survival (PFS) based on metastatic sites at baseline. Kaplan-Meier curves for patients receiving CDK4/6 inhibitor plus endocrine therapy are stratified by the presence or absence of **(A)** liver metastasis, **(B)** bone metastasis, **(C)** lung metastasis, and **(D)** lymph node metastasis. The number of patients at risk in each subgroup at specified time points is shown below the corresponding panel.

We also evaluated whether metastatic site influenced treatment effectiveness. Patients with liver metastasis and/or bone metastasis had significantly shorter PFS (Figures 8A,B; P = 0.0001 and P = 0.0342, respectively). In contrast, lung metastasis and lymph node metastasis did not significantly affect PFS (all P > 0.05, Figures 8C,D).

2. For overall survival (OS), the median follow-up periods for the three CDK4/6 inhibitor plus endocrine therapy (ET) groups were 25.8, 11.4, and 24.6 months, respectively. During the follow-up period, there was one death in the dalpiciclib group, two in the abemaciclib group, and three in the palbociclib group. At the data cut-off, the OS data were still immature and were not analyzed due to an insufficient number of events.

### Safety and tolerability

Cyclin-dependent kinase 4/6 inhibitors (CDK4/6i) represented a milestone in the treatment of breast cancer. Given the distinct adverse effect profiles of different CDK4/6 inhibitors—palbociclib, abemaciclib, and dalpiciclib—careful evaluation of their toxicities was essential for informing clinical decision-making. However, the lack of head-to-head clinical trials underscored the need for comprehensive safety data to compare the risks of adverse events (AEs) among these agents.

According to the PALOMA-2 and PALOMA-3 trials, the most common grade 3 or 4 AEs associated with palbociclib were neutropenia, leukopenia, and anemia (Rugo et al., 2019; Cristofanilli et al., 2016). In the MONARCH-2 and MONARCH-3 studies, abemaciclib most frequently led to grade 3 or 4 diarrhea, neutropenia, leukopenia, nausea, and fatigue (Goetz et al., 2017). Data from the DAWNA-1 and DAWNA-2 trials indicated that the most common grade 3 or 4 AEs for dalpiciclib included neutropenia and leukopenia (Xu et al., 2021; Zhang et al., 2023). In our study, as summarized in Table 3, the safety profiles and detailed AEs were consistent with these previously reported literature.

**TABLE 3:** Treatment-related adverse effects.

Adverse event, n (%)	Palbociclib + ET (n = 138)			Abemaciclib + ET (n = 119)			Dalpiciclib + ET (n = 84)		
	Grade 1–2	Grade 3	Grade 4	Grade 1–2	Grade 3	Grade 4	Grade 1–2	Grade 3	Grade 4
Neutropenia	16 (11.60%)	5 (3.62%)	5 (3.62%)	12 (10.08%)	2 (1.68%)	1 (0.84%)	11 (13.10%)	9 (10.71%)	3 (3.57%)
Leukopenia	15 (10.87%)	7 (5.07%)	4 (2.90%)	13 (10.92%)	0	0	18 (21.43%)	5 (5.95%)	2 (2.38%)
Anemia	8 (5.80%)	0	0	3 (2.52%)	0	0	9 (10.71%)	1 (1.19%)	0
Thrombocytopenia	9 (6.52%)	2 (1.45%)	1 (0.72%)	5 (4.20%)	1 (0.84%)	0	11 (13.10%)	0	1 (1.19%)
Fatigue	0	0	0	9 (7.56%)	0	0	0	0	0
Nausea	0	0	0	3 (2.52%)	0	0	0	0	0
Vomiting	0	0	0	3 (2.52%)	0	0	0	0	0
AST/ALT increased	2 (1.45%)	0	0	3 (2.52%)	0	0	10 (11.90%)	0	0
Cr/BUN increased	0	0	0	1 (0.84%)	0	0	1 (1.19%)	0	0
Diarrhea	0	0	0	29 (24.37%)	0	0	1 (1.19%)	0	0
Rash	0	0	0	5 (4.20%)	0	0	4 (4.76%)	0	0
Oral mucositis	0	0	0	0	0	0	2 (2.38%)	0	0
Bilirubin increased	0	0	0	0	0	0	1 (1.19%)	0	0
PAC	0	0	0	0	0	0	1 (1.19%)	0	0
Pneumonia	0	0	0	1 (0.84%)	0	0	1 (1.19%)	0	0

AST, aspartate aminotransferase; ALT, alanine aminotransferase; PAC, premature atrial contractions. Neutropenia is any event having a preferred term that equals to Neutropenia or Neutrophil count decreased. Leukopenia is any event having a preferred term that equals to Leukopenia or White blood cell count decreased. Anemia is any event having a preferred term that equals to Anaemia or Haematocrit decreased or Haemoglobin decreased. Thrombocytopenia is any event having a preferred term that equals to Platelet count decreased or Thrombocytopenia. Rash is any event having a preferred term that equals to Dermatitis or Dermatitis acneiform or Rash or Rash erythematous or Rash maculo-papular or Rash papular or Rash pruritic or Toxic skin eruption.

Palbociclib was primarily associated with hematologic toxicities. Leukopenia occurred in 26 of 138 patients (grade 1: 8; grade 2: 7; grade 3: 7; grade 4: 4). Neutropenia was observed in 26 patients (grade 1: 10; grade 2: 6; grade 3: 5; grade 4: 5). Anemia was reported in 8 cases (grade 1: 2; grade 2: 6), and thrombocytopenia in 12 cases (grade 1: 3; grade 2: 6; grade 3: 2; grade 4: 1). Hepatic function abnormalities, manifested as elevated ALT and AST, were noted in 2 patients, both grade 1.

Abemaciclib-related AEs predominantly included diarrhea and hematologic toxicities. Leukopenia was reported in 13 of 119 patients (grade 1: 9; grade 2: 4). Neutropenia occurred in 15 patients (grade 1: 8; grade 2: 4; grade 3: 2; grade 4: 1). Anemia was observed in 3 patients (all grade 1), and thrombocytopenia in 6 patients (grade 1: 2; grade 2: 3; grade 3: 1). Elevated ALT and AST were noted in 3 patients, all grade 1. One patient experienced grade 1 renal impairment. Diarrhea occurred in 29 patients (grade 1: 26; grade 2: 3). Other AEs included rash (5 cases, all grade 1), fatigue (9 cases, all grade 1), nausea/vomiting (3 cases, all grade 1), and pneumonia (1 case, grade 2).

Dalpiciclib-induced AEs were mainly hematologic toxicities. Leukopenia was reported in 25 of 84 patients (grade 1: 3; grade 2: 15; grade 3: 5; grade 4: 2). Neutropenia occurred in 23 patients (grade 1: 4; grade 2: 7; grade 3: 9; grade 4: 3). Anemia was observed in 10 patients (grade 1: 3; grade 2: 6; grade 3: 1), and thrombocytopenia in 12 patients (grade 1: 3; grade 2: 8; grade 4: 1). Hepatic function abnormalities (elevated ALT/AST) were noted in 10 patients (grade 1: 4; grade 2: 6). One case each of grade 1 renal impairment and grade 2 hyperbilirubinemia was reported. Other AEs included diarrhea (1 case, grade 1), rash (4 cases, grade 1), oral mucositis (2 cases, grade 1), atrial premature beats (1 case, grade 2), and pneumonia (1 case, grade 2).

Safety was assessed in all patients who received at least one dose of CDK4/6i combined with endocrine therapy. Dalpiciclib and palbociclib were associated with a higher risk of hematologic toxicities, whereas abemaciclib posed the highest risk of gastrointestinal toxicity, particularly diarrhea. Hematologic toxicities and hepatotoxicity were common across palbociclib, abemaciclib, and dalpiciclib. Renal toxicities, rash, and diarrhea were observed with abemaciclib and dalpiciclib. Dalpiciclib was associated with oral mucositis, hyperbilirubinemia, atrial premature beats, and pneumonia. AEs related to abemaciclib included fatigue, nausea, vomiting, and pneumonia. These findings offer valuable insights for the selection of CDK4/6 inhibitors. No treatment-related deaths occurred. Overall, the combination of CDK4/6 inhibitors and endocrine therapy exhibited a favorable safety profile during follow-up in patients with advanced breast cancer.

### Potential mutation tests

Anti-estrogen therapies, frequently used in combination with a CDK4/6 inhibitor, constituted the first-line standard of care for advanced HR + breast cancer. Nevertheless, treatment resistance invariably occurred, driven by either ER-dependent or ER-independent mechanisms promoting tumor progression. Novel endocrine agents—such as next-generation oral SERDs and PROTACs—show potential effectiveness, particularly in ESR1-mutant breast cancer. Additionally, tumors carrying driver mutations in pathways like PI3K/AKT/mTOR or PTEN might respond to new regimens combining endocrine and pathway-specific inhibitors (Lloyd et al., 2024). In this study, next-generation sequencing (NGS) was performed on patient samples to explore potential genomic correlation. Among those with evaluable NGS results, the palbociclib plus ET group included one case with a PIK3CA mutation (Exon20: c.3140A>G, p. H1047R) and one with a TP53 mutation (Exon8: c.853G>A, p. E285K). In the abemaciclib plus ET group, one patient carried a PIK3CA mutation (Exon9: c.1633G>A, p. E545K), one had a PTEN mutation (Exon5: c.324del, p. D109Tfs4), one exhibited an AKT1 mutation (Exon3: c.49G>A, p. E17K), and two showed PALB2 alterations (Exon4: c.1142_1143del, p. L381Qfs19; c.2835-1G>C). In the dalpiciclib plus ET group, only one TP53 mutation (Exon8: c.853G>A, p. E285K) was detected. Given the limited number of patients with detectable genomic alterations, it remains challenging to assess whether specific mutations influenced the effectiveness or prognosis of CDK4/6 inhibitor plus ET treatment in this study.

## Discussion

HR+/HER2− breast cancer accounts for more than two-thirds of all breast cancer cases. Endocrine therapy (ET) serves as the cornerstone of systemic treatment for this subtype. Since metastatic breast cancer is incurable, there is an urgent clinical need to improve therapeutic outcomes, optimize treatment strategies and drug selection, and prolong survival for these patients. In recent years, multiple studies have demonstrated the clinical effectiveness of combining cyclin-dependent kinase 4/6 (CDK4/6) inhibitors with ET in metastatic breast cancer. Key phase III trials, including PALOMA-2, PALOMA-3, MONARCH-2, MONARCH-3, MONALEESA-2, MONALEESA-3, MONALEESA-7, DAWNA-1, and DAWNA-2, have consistently shown that adding a CDK4/6 inhibitor to ET improves outcomes for patients with HR+/HER2− MBC, establishing this combination as a standard first-line treatment based on significant progression-free survival (PFS) benefits (Gradishar et al., 2023). Furthermore, updated results from MONARCH-2 and MONALEESA-2, -7 indicated that abemaciclib or ribociclib plus ET significantly prolonged OS compared with ET alone. Current treatment decisions regarding the use of CDK4/6 inhibitors, such as palbociclib, abemaciclib, dalpiciclib, and ribociclib, in combination with endocrine therapy are often guided by differences in their toxicity profiles. It is commonly assumed that patients who are intolerant to one CDK4/6 inhibitor may experience fewer adverse effects to another. However, this approach to drug selection lacks scientific evidence. Direct head-to-head clinical trials comparing the effectiveness of different CDK4/6 inhibitors combined with specific endocrine therapies in this population are lacking. Thus, it is essential to determine which specific CDK4/6 inhibitor combined with which endocrine agent yields the best effectiveness and prognosis in patients with HR+/HER2− MBC.

Aromatase inhibitors (AIs; letrozole, anastrozole, and exemestane) inhibit estrogen synthesis in postmenopausal women and reduce estrogen receptor (ER) signaling by lowering estrogen levels. Selective estrogen receptor degraders (SERDs; fulvestrant) directly bind to ER, block ER signaling, and target ER for degradation; most SERDs act as complete antagonists (Chen et al., 2022). In the first-line treatment of advanced breast cancer with CDK4/6 inhibitors combined with ET, both CDK4/6 inhibitor plus AI and CDK4/6 inhibitor plus SERD have been supported by clinical trial data demonstrating prolonged PFS. In this study, we compared the effectiveness and PFS outcomes of three CDK4/6 inhibitors—palbociclib, abemaciclib, and dalpiciclib—combined with AIs (anastrozole, letrozole, and exemestane) or with the SERD fulvestrant. To determine the optimal partner of CDK4/6i plus ET, the predictive biomarkers were also evaluated. Ribociclib was not included due to its later approval in China.

As shown in Figure 2, no significant differences in ORR or CBR were observed among the three CDK4/6 inhibitors. Due to the limited PFS events, the median PFS for the palbociclib, abemaciclib, and dalpiciclib groups was 25.3 months, not reached (NR), and 36.0 months, respectively (Figure 3). In the MONARCH-2 trial, abemaciclib plus fulvestrant significantly extended the median PFS (mPFS) to 16.4 months in patients whose breast cancer had progressed on prior endocrine therapy. Similarly, the MONARCH-3 study reported an mPFS of 28.18 months with abemaciclib plus a nonsteroidal aromatase inhibitor (NSAI; either anastrozole or letrozole) in treatment-naïve postmenopausal women with HR+/HER2− metastatic breast cancer (MBC). Prior to receiving CDK4/6 inhibitor (CDK4/6i) plus ET, first-line chemotherapy had been administered to 21.7% of patients in the palbociclib group, 50.4% in the abemaciclib group, and 19.0% in the dalpiciclib group. More than half of the patients treated with abemaciclib in our cohort had previously received chemotherapy in the metastatic setting, reflecting clinical practice patterns at the time, wherein patients with higher disease burden often underwent chemotherapy before transitioning to ET combined with a CDK4/6 inhibitor. In addition, given the relatively small number of PFS events (17 events out of 119 cases) in the abemaciclib combination group, even after statistical adjustments, further follow-up data are still required to draw a definitive conclusion regarding its superiority over the palbociclib combination regimen. For PFS, statistical analysis revealed that the abemaciclib group had longer progression-free survival compared to the palbociclib group (abemaciclib + ET vs. palbociclib + ET, p = 0.0426, hazard ratio [95% confidence interval]: 0.54 [0.30–0.98]). However, it should be noted that the short follow-up duration and the very low number of events may introduce bias into the time-to-event analyses, complicating the comparative interpretation of PFS outcomes. Moreover, the median PFS not being reached in the abemaciclib group is likely attributable, at least in part, to data immaturity rather than the treatment effect alone. Based on the preliminary findings of this study, a more rigorously designed prospective, randomized, double-blind, placebo-controlled Phase III trial can further minimize bias and confounding factors in the future.

Moreover, within the three CDK4/6 inhibitor treatment groups, 42.0% (58/138), 26.1% (31/119), and 28.6% (24/84) of patients received treatment with a CDK4/6 inhibitor plus fulvestrant, while the remaining patients received one of the three different AIs. A higher proportion of patients received fulvestrant as first-line endocrine therapy in the palbociclib combination group compared to other CDK4/6 inhibitor groups, suggesting clinician bias toward its use in cases with suspected AI resistance. This might account for the inferior progression-free survival (PFS) observed with palbociclib, though this hypothesis required validation in future studies. In the comparison of palbociclib combined with AIs versus fulvestrant, palbociclib plus fulvestrant showed a trend toward superior PFS compared with palbociclib plus AIs, although the difference did not reach statistical significance. No significant PFS differences were observed between AIs and fulvestrant in the other two CDK4/6 inhibitor groups (Figure 4). Forest plot results indicated that, based on the current sample size and without distinguishing between specific CDK4/6 inhibitors used, although statistical significance was not reached, a consistent trend toward PFS benefit was observed in the fulvestrant-based combination therapy group (Figure 5). This trend may be confirmed with further expansion of the enrolled population in future studies.

In this study, no significant association was observed between PFS following CDK4/6 inhibitor plus endocrine therapy and the status of key molecular biomarkers, including hormone receptor (HR) expression, HER2 status (negative vs. low), Ki67 expression or tumor-infiltrating lymphocytes (TILs) (Figure 6). A more rigorous and well-designed prospective study should be conducted to investigate potential biomarkers capable of predicting PFS benefit from combination therapy.

All enrolled patients received combination therapy with a CDK4/6 inhibitor and ET. Consistent with the landmark studies, our patient enrollment criteria also allowed for the inclusion of individuals who had not received prior endocrine therapy in the advanced metastatic setting but may have undergone first-line chemotherapy. Therefore, in our study, the term “first-line treatment” refers to the use of CDK4/6 inhibitors combined with ET following possible prior first-line chemotherapy. This context includes scenarios where patients progressed after chemotherapy and subsequently received CDK4/6i plus ET, as well as cases where chemotherapy achieved stable disease or good control, and CDK4/6i plus ET was then administered as maintenance therapy. Prior to initiating combination treatment, 21.7%, 50.4%, and 19.0% of patients in the three CDK4/6i + ET groups had received common chemotherapeutic agents—such as taxanes or anthracyclines—as first-line systemic therapy. Our findings indicated that even after prior first-line chemotherapy, patients with MBC could still achieve PFS benefits from CDK4/6 inhibitor plus ET. Visceral crisis was defined as acute or severe organ dysfunction caused by extensive visceral metastasis, requiring rapid and effective intervention. Although none of the patients in this study presented with visceral crisis, the majority had visceral metastases in one or more sites (including liver, lung, brain, bone marrow, peritoneum, pleura, and adrenal glands), with 61.6%, 57.3%, and 65.5% of patients having visceral metastases at the time of CDK4/6i + ET initiation. Therefore, especially for patients with multi-organ visceral involvement, chemotherapy was not the only option. Our results demonstrated that first-line CDK4/6 inhibitor combined with ET could still provide survival benefits in this setting, which aligned with the findings of the RIGHT Choice trial (Lu et al., 2024). Compared with chemotherapy, CDK4/6i plus ET was associated with relatively milder side effects and better tolerability, making it a favorable option for maintaining or improving patients’ quality of life. Our findings indicated that the presence of liver or bone metastases was independently associated with shorter progression-free survival (PFS), even under uniform treatment regimens. This highlighted the need for personalized treatment strategies to improve outcomes in these subpopulations. Given the multiple comparisons among different CDK4/6 inhibitors and endocrine therapies, the results should not be interpreted as confirmatory.

Safety monitoring indicated that the treatment was well tolerated, with no new safety signals identified in this study. Consistent with previous reports, the most common adverse events (AEs) were grade 1–2 in severity. Hematologic toxicities were frequently observed, while gastrointestinal AEs—particularly diarrhea—were most common in the abemaciclib combination group. In this study, we observed that the proportion of CDK4/6 inhibitor-treated patients who exhibited declines in hematological parameters was lower than the data reported in pivotal clinical trials. This may be attributed to the concomitant use of oral leukopoietic agents during treatment. Given the lower incidence of hematological adverse events compared with literature reports, the proportion of patients requiring dose reduction or discontinuation of CDK4/6 inhibitors due to hematological adverse events was also reduced in our cohort. Only 17.39%, 3.36%, and 25% of patients in the palbociclib, abemaciclib, and dalpiciclib combination groups, respectively, experienced grade 3–4 treatment-related AEs. All AEs resolved with or without standard management and were effectively controlled through medication and dose adjustments without compromising effectiveness. No new AEs signals were found in the study. The overall AE profile was acceptable, supporting the use of CDK4/6i plus ET as a viable treatment strategy and reinforcing clinician confidence in its application.

Although this study has provided an in-depth exploration and analysis of the effectiveness and safety of combining three types of CDK4/6 inhibitors with endocrine therapy for advanced HR+/HER2− breast cancer, certain limitations remain. While multivariable Cox regression was performed, residual confounding may still exist due to the retrospective design and baseline imbalances. Propensity score-based methods (e.g., matching or inverse probability weighting) could further improve balance between groups. However, considering the sample size and the exploratory nature of this study, such adjustments may not be suitable. Future prospective studies are warranted to more clearly address these questions in clinical practice. Given the heterogeneity and aggressiveness of breast cancer, multiple therapeutic approaches and combination regimens are essential to improve outcomes. Based on our observational and exploratory designed real-world study, the combination of CDK4/6 inhibitors and ET provides clinical benefits with a manageable safety profile, offering a valuable treatment strategy for patients with advanced HR+/HER2− breast cancer.

## Data Availability

The original contributions presented in the study are included in the article/supplementary material, further inquiries can be directed to the corresponding authors.
