# Supplementary figures and images for: Comparative effectiveness of CDK4/6 inhibitor plus endocrine therapy combinations in HR-positive, HER2-negative metastatic breast cancer: the inspiration 01 study
Source: Front Pharmacol. 2026 Apr 13;17:1757379. doi: 10.3389/fphar.2026.1757379 (PMC13111907; doi:10.3389/fphar.2026.1757379)

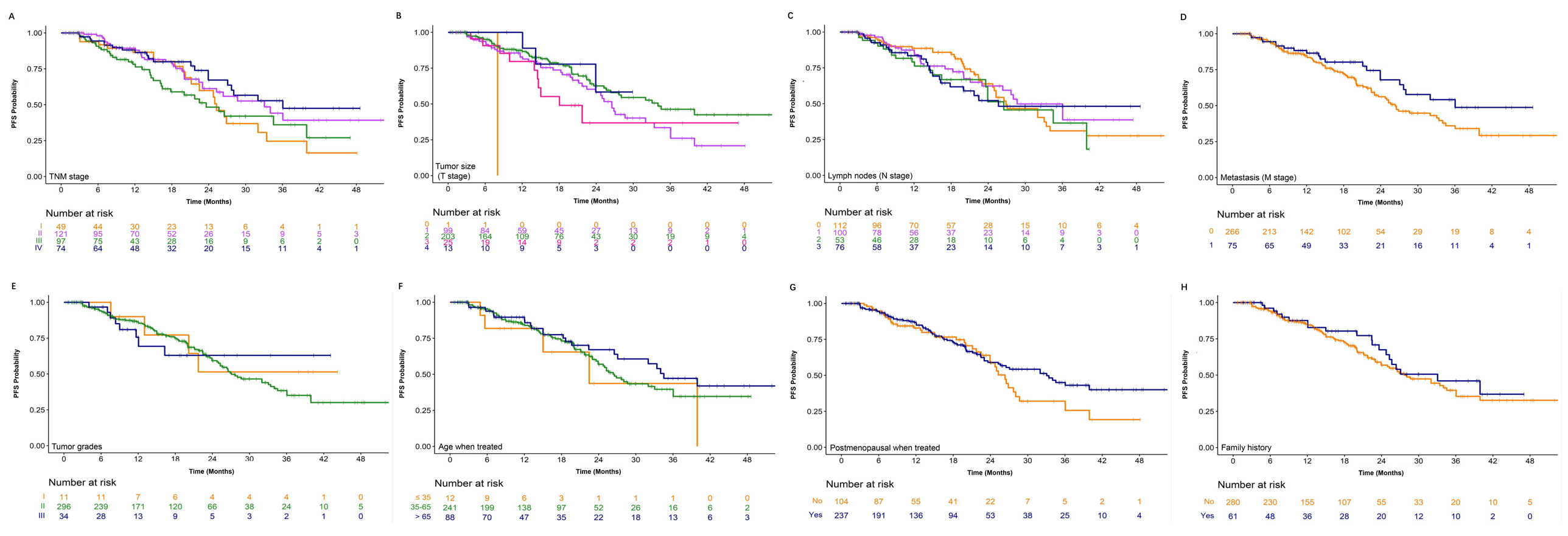

Supplement: Supplementary file 1 [file Image2.tif]

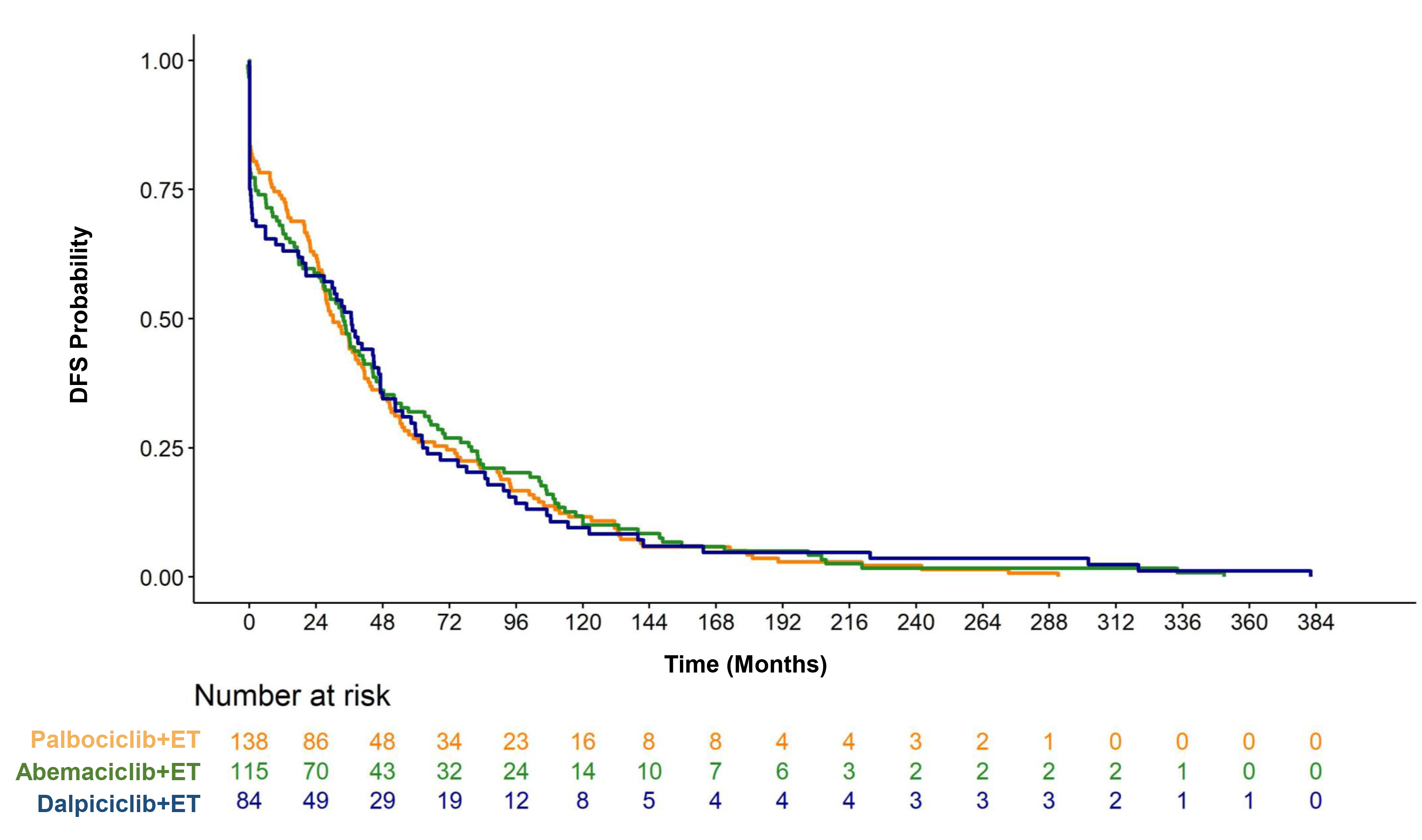

Supplement: Supplementary file 3 [file Image1.tif]
