# Supplementary material for: Comparative effectiveness of CDK4/6 inhibitor plus endocrine therapy combinations in HR-positive, HER2-negative metastatic breast cancer: the inspiration 01 study
Source: Front Pharmacol. 2026 Apr 13;17:1757379. doi: 10.3389/fphar.2026.1757379 (PMC13111907; doi:10.3389/fphar.2026.1757379)

Figure S1. Survival curves showed no difference among the three CDK4/6i (palbociclib, abemaciclib, and dalpiciclib) plus ET groups in DFS (P > 0.05).

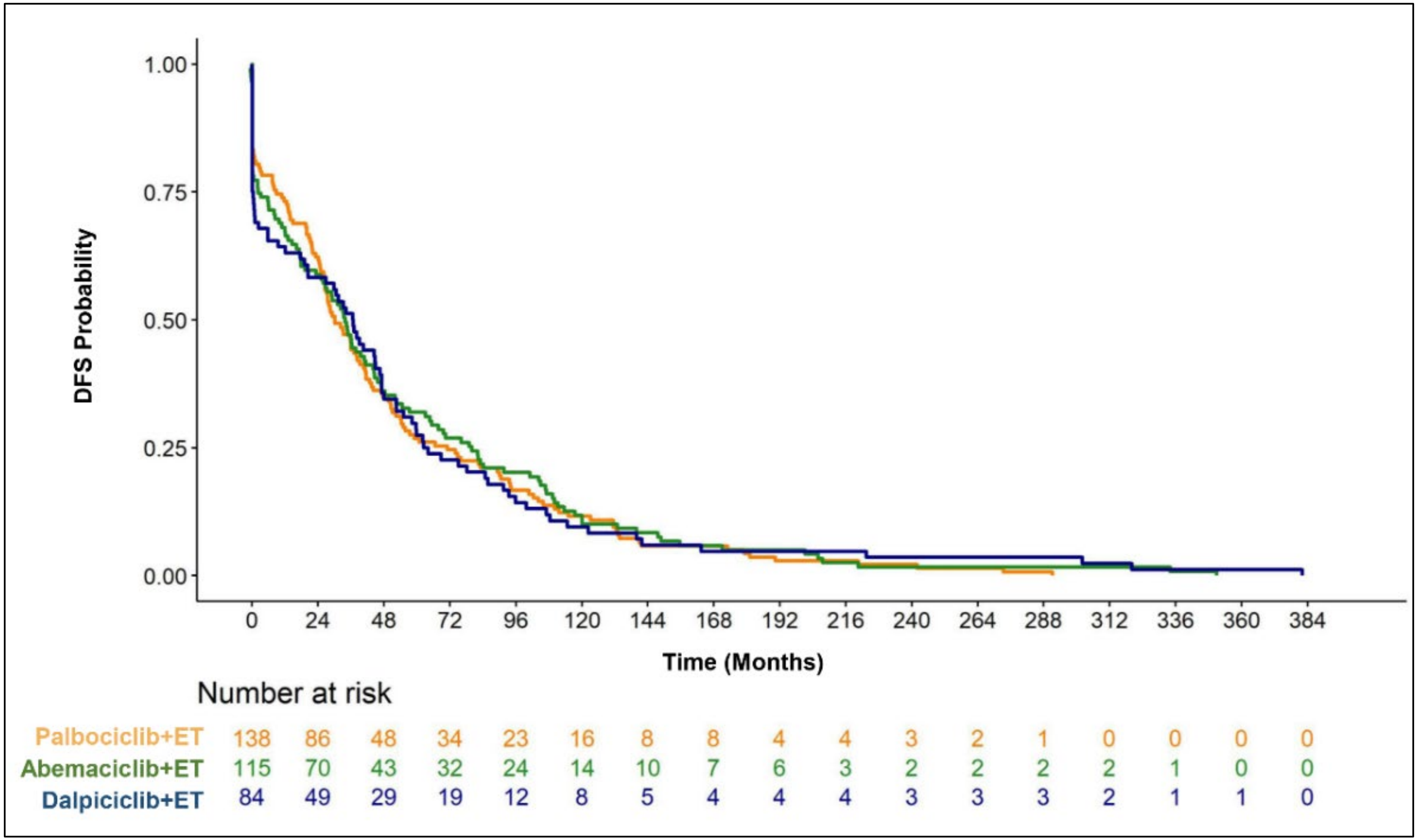

Figure S2. Kaplan-Meier curves for progression-free survival (PFS) in patients with HR+/HER2- metastatic breast cancer, stratified by specific clinical signature (TNM stage, tumor size, lymph nodes status, metastasis, tumor grade, age, menopausal status and family history). The number of patients at risk in each subgroup over time is listed in the corresponding table.

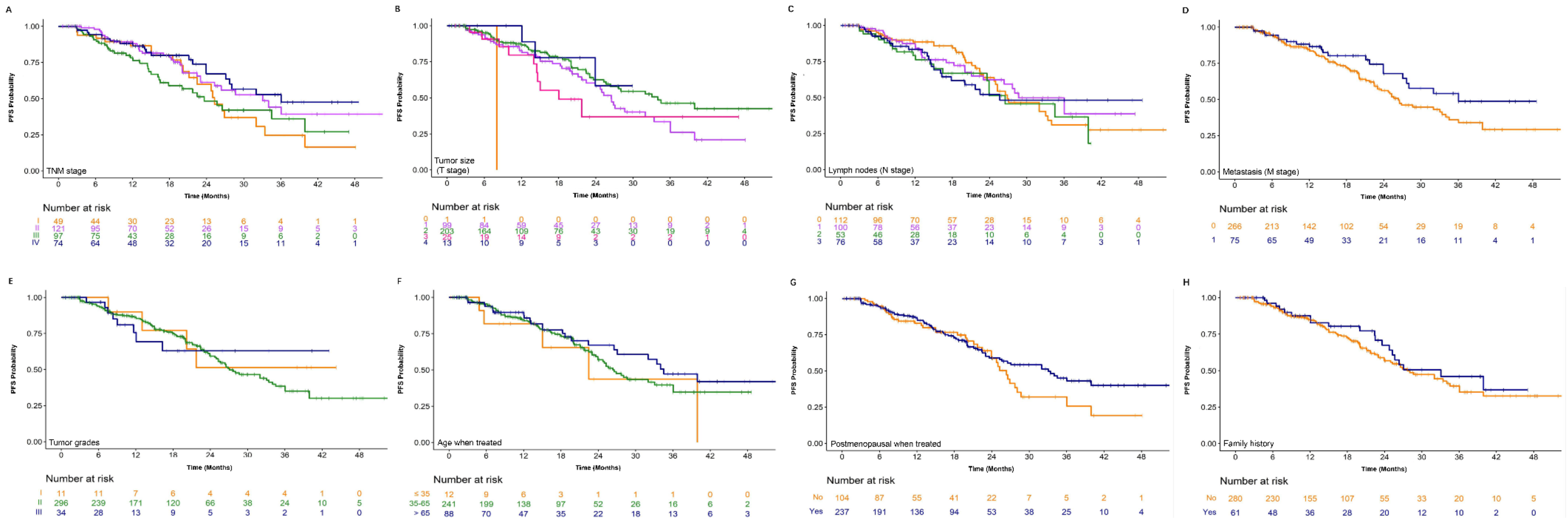

Supplement: Supplementary file 2 [file Supplementaryfile1.pdf]
